# Supplementary material for: Playing the role of weak clique property in link prediction: A friend recommendation model
Source: Sci Rep. 2016 Jul 21;6:30098. doi: 10.1038/srep30098 (PMC4954950; doi:10.1038/srep30098)
Supplement: Supplementary Information [file srep30098-s1.pdf]

Supplementary Information for

Playing the role of weak clique property in link prediction: A friend recommendation model

Chuang Ma,[1] Tao Zhou,[2] and Hai-Feng Zhang[1,3,4]

[1] *School of Mathematical Science, Anhui University, Hefei 230601, China*
[2] *Web Sciences Center, University of Electronic Science and Technology of China, Chengdu 610054, China*
[3] *Key Laboratory of Computer Network and Information Integration (Southeast University), Ministry of Education, 211189, P. R. China*
[4] *Center of Information Support &Assurance Technology, Anhui University, Hefei 230601, China*

(Dated: May 19, 2016)

TYPICAL PWCS PHENOMENON

To confirm that the PWCS phenomenon typically exists in real networks, we also calculate the values of P_1, P_2 and P_3 in other 15 real networks, including networks with small sizes and large sizes. The definitions of P_1, P_2 and P_3 are given in Eqs. (1), (2) and (3) in main text, respectively. We say that the PWCS phenomenon exists in the network if $P_1 > P_2$ and $P_1 > P_3$. Furthermore, the PWCS phenomenon is significant if $P_1 > P_2 > P_3$, otherwise, the PWCS phenomenon is weak when $P_1 > P_3 \geq P_2$. From Tab. S1, we can find that $P_1 > P_2$ and $P_1 > P_3$ in all 15 real networks, and there are 6 networks having significant PWCS phenomenon (labelled in blue color).

COMPARISON OF FOUR INDICES IN NETWORKS WITH LARGE SIZES

On the one hand, to prove that our FR index is a local similarity index and can deal with networks with large sizes; on the other hand, to compare the effects of CN, AA, RA and our FR indices on Precision. In Fig. S1 we plot Precision as a function of L for the four indices in six real networks. The network size spans from $N = 16706$ to $N = 325729$ (The basic information for the six networks is presented in Tab. S2). In general, the results in Fig. S1 indicate that our proposed FR index has a better performance for a large range of L.

[1] Newman M E J. The structure of scientific collaboration networks, Proceedings of the National Academy of Sciences, 2001: 98(2): 404-409.

[2] Leskovec J, Kleinberg J, Faloutsos C. Graphs over time: densification laws, shrinking diameters and possible explanations, Proceedings of the eleventh ACM SIGKDD international conference on Knowledge discovery in data mining. ACM, 2005: 177-187.

Table S1: The values of P_1, P_2 and P_3 in 15 real networks are reported. Results in networks with significant PWCS, i.e., $P_1 > P_2 > P_3$ are shown in blue color. N is the size of network.

Network	N	P_1	P_2	P_3
Dolphins	62	0.4678	0.1920	0.2760
Email	1133	0.3088	0.1107	0.0902
Facebook	334	0.7101	0.2311	0.3089
Karate	34	0.5417	0.1587	0.2389
Kohonen	4469	0.06	0.0238	0.0191
Lesmis	77	0.8818	0.2828	0.4397
SciMet	3084	0.1814	0.0746	0.0538
Adjacencies	112	0.1684	0.1679	0.1078
Football	115	0.9292	0.2082	0.4206
Hep	8361	0.5846	0.1682	0.2995
Y2H	2112	0.3397	0.0728	0.0184
PGP	10680	0.5439	0.0760	0.1170
SmaGri	1059	0.1561	0.0615	0.0531
Astro-phys	16706	0.5973	0.1405	0.3042
Hep-th	27240	0.1703	0.0694	0.0874

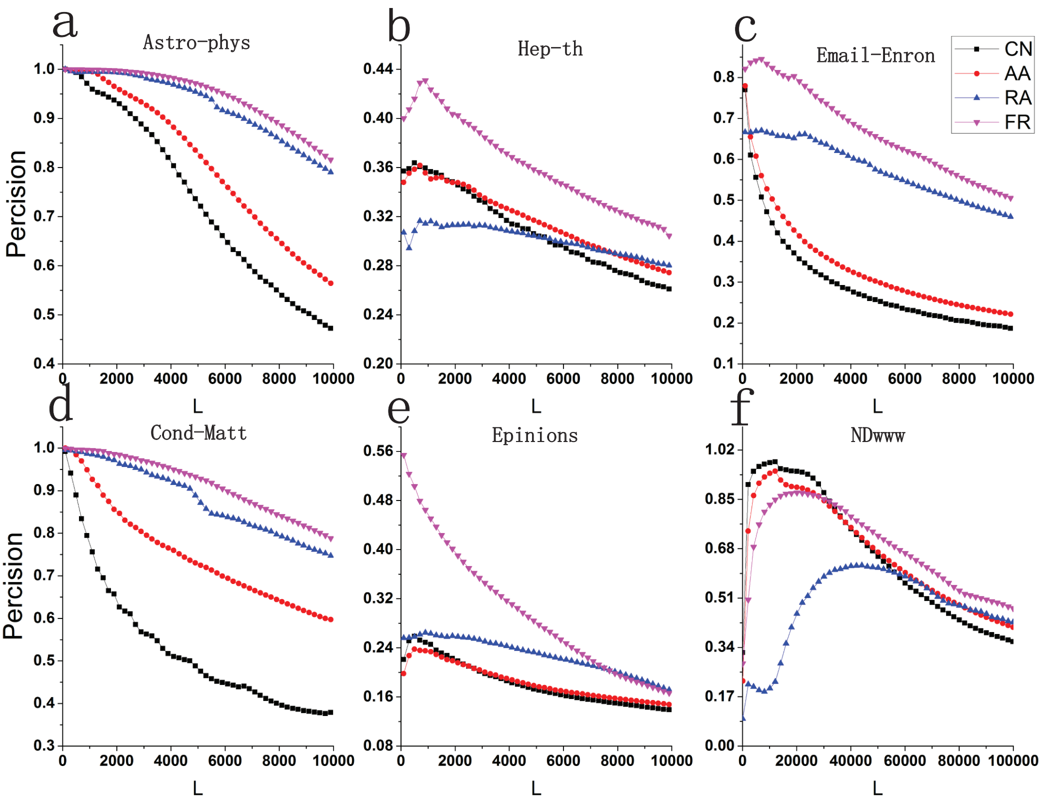

Figure S1: Precision as a function of L is compared in six real networks for CN, AA, RA and FR indices.

Table S2: Basic information about the six real networks used in Fig. S1. N is the size of network.

Network	N	Reference
Astro-phys	16706	[1]
Astro-phys	27770	[2]
Email-Enron	36692	[3]
Cond-Matt	40421	[1]
Epinions	75879	[4]
NDwww	325729	[5]

[3] Leskovec J, Lang K J, Dasgupta A, et al. Community structure in large networks: Natural cluster sizes and the absence of large well-defined clusters, Internet Mathematics, 2009, 6(1): 29-123.

[4] Richardson M, Agrawal R, Domingos P. Trust management for the semantic web, The Semantic Web-ISWC 2003. Springer Berlin Heidelberg, 2003: 351-368.

[5] Albert R, Jeong H, Barabási A L. Internet: Diameter of the world-wide web. Nature, 1999, 401(6749): 130-131.
